# CYP6 P450 Enzymes and *ACE-1* Duplication Produce Extreme and Multiple Insecticide Resistance in the Malaria Mosquito *Anopheles gambiae*

**DOI:** 10.1371/journal.pgen.1004236

**Published:** 2014-03-20

**Authors:** Constant V. Edi, Luc Djogbénou, Adam M. Jenkins, Kimberly Regna, Marc A. T. Muskavitch, Rodolphe Poupardin, Christopher M. Jones, John Essandoh, Guillaume K. Kétoh, Mark J. I. Paine, Benjamin G. Koudou, Martin J. Donnelly, Hilary Ranson, David Weetman

**Affiliations:** 1 Vector Biology Department, Liverpool School of Tropical Medicine, Pembroke Place, Liverpool, United Kingdom; 2 Centre Suisse de Recherches Scientifiques en Côte d'Ivoire, Abidjan, Cote d'Ivoire; 3 Institut Régional de Santé Publique/Université d ‘Abomey-Calavi, Ouidah, Bénin; 4 Biology Department, Boston College, Chestnut Hill, Massachusetts, United States of America; 5 Department of Entomology and Wildlife, University of Cape Coast, Cape Coast, Ghana; 6 Unité de Recherche en Ecotoxicologie (URET), Faculty of Sciences, Université de Lomé, Lomé, Togo; 7 Centre for Neglected Tropical Diseases, Liverpool School of Tropical Medicine, Pembroke Place, Liverpool, United Kingdom; 8 Université Nangui-Abrogoua, UFR Sciences de la Nature, Abidjan, Côte d'Ivoire; 9 Malaria Programme, Wellcome Trust Sanger Institute, Hinxton, Cambridge, United Kingdom; University of Michigan, United States of America

## Abstract

Malaria control relies heavily on pyrethroid insecticides, to which susceptibility is declining in *Anopheles* mosquitoes. To combat pyrethroid resistance, application of alternative insecticides is advocated for indoor residual spraying (IRS), and carbamates are increasingly important. Emergence of a very strong carbamate resistance phenotype in *Anopheles gambiae* from Tiassalé, Côte d'Ivoire, West Africa, is therefore a potentially major operational challenge, particularly because these malaria vectors now exhibit resistance to multiple insecticide classes. We investigated the genetic basis of resistance to the most commonly-applied carbamate, bendiocarb, in *An. gambiae* from Tiassalé. Geographically-replicated whole genome microarray experiments identified elevated P450 enzyme expression as associated with bendiocarb resistance, most notably genes from the CYP6 subfamily. P450s were further implicated in resistance phenotypes by induction of significantly elevated mortality to bendiocarb by the synergist piperonyl butoxide (PBO), which also enhanced the action of pyrethroids and an organophosphate. *CYP6P3* and especially *CYP6M2* produced bendiocarb resistance via transgenic expression in *Drosophila* in addition to pyrethroid resistance for both genes, and DDT resistance for *CYP6M2* expression. CYP6M2 can thus cause resistance to three distinct classes of insecticide although the biochemical mechanism for carbamates is unclear because, in contrast to CYP6P3, recombinant CYP6M2 did not metabolise bendiocarb *in vitro*. Strongly bendiocarb resistant mosquitoes also displayed elevated expression of the acetylcholinesterase *ACE-1* gene, arising at least in part from gene duplication, which confers a survival advantage to carriers of additional copies of resistant *ACE-1* G119S alleles. Our results are alarming for vector-based malaria control. Extreme carbamate resistance in Tiassalé *An. gambiae* results from coupling of over-expressed target site allelic variants with heightened CYP6 P450 expression, which also provides resistance across contrasting insecticides. Mosquito populations displaying such a diverse basis of extreme and cross-resistance are likely to be unresponsive to standard insecticide resistance management practices.

## Author Summary

Malaria control depends heavily on only four classes of insecticide to which *Anopheles* mosquitoes are increasingly resistant. It is important to manage insecticide application carefully to minimise increases in resistance, for example by using different compounds in combination or rotation. Recently, mosquitoes resistant to all available insecticides have been found in Tiassalé, West Africa, which could be problematic for resistance management, particularly if common genetic mechanisms are responsible (‘cross-resistance’). Tiassalé mosquitoes also exhibit extreme levels of resistance to the two most important classes, pyrethroids and carbamates. We investigated the genetic basis of extreme carbamate resistance and cross-resistance in Tiassalé, and the applicability of results in an additional population from Togo. We find that specific P450 enzymes are involved in both extreme and cross-resistance, including one, CYP6M2, which can cause resistance to three insecticide classes. However, amplification of a mutated version of the gene which codes for acetycholinesterase, the target site of both the carbamate and organophosphate insecticides, also plays an important role. Mechanisms involved in both extreme resistance and cross resistance are likely to be very resilient to insecticide management practices, and represent an alarming scenario for mosquito-targeted malaria control.

## Introduction

Malaria mortality has decreased substantially in sub-Saharan Africa over the last decade, attributed in part to a massive scale-up in insecticide-based vector control interventions [1]. As the only insecticide class approved for treatment of bednets (ITNs) and the most widely used for indoor residual spraying (IRS), pyrethroids are by far the most important class of insecticides for control of malaria vectors [2]. Unfortunately pyrethroid resistance is now widespread and increasing in the most important malaria-transmitting *Anopheles* species [3]–[5] and catastrophic consequences are predicted for disease control if major pyrethroid failure occurs [6]. With no entirely new insecticide classes for public health anticipated for several years [5], [6] preservation of pyrethroid efficacy is critically dependent upon strategies such as rotation or combination of pyrethroids with just three other insecticide classes, organochlorines, carbamates and organophosphates [6], [7]. In addition to logistical and financial issues, insecticide resistance management suffers from knowledge-gaps concerning mechanisms causing cross-resistance between available alternative insecticides, and more, generally how high-level resistance arises [8]. With strongly- and multiply-resistant phenotypes documented increasingly in populations of the major malaria vector *Anopheles gambiae* in West Africa [9]–[13] such information is urgently required.

Of the four classes of conventional insecticide licensed by the World Health Organisation (WHO), pyrethroids and DDT (the only organochlorine) both target the same *para*-type voltage-gated sodium channel (VGSC). This creates an inherent vulnerability to cross-resistance via mutations in the *VGSC* target site gene [14]–[16], which are now widespread in *An. gambiae* [5]. In contrast, carbamates and organophosphates cause insect death by blocking synaptic neurotransmission via inhibition of acetylcholinesterase (AChE), encoded by the *ACE-1* gene in *An. gambiae*. Consequently, target site mutations in the *VGSC* gene producing resistance to pyrethroids and DDT will not cause cross-resistance to carbamates and organophosphates. The carbamate bendiocarb is being used increasingly for IRS [17], [18], and has proved effective in malaria control programs across Africa targeting pyrethroid- or DDT-resistant *An. gambiae* [18]–[20]. A single nucleotide substitution of glycine to serine at codon position 119 (*Torpedo* nomenclature; G119S) in the *ACE-1* gene, which causes a major conformational change in AChE, has arisen multiple times in culicid mosquitoes [21], [22], and is found in *An. gambiae* throughout West Africa [23]–[25]. The G119S mutation can produce carbamate or organophosphate resistance [26] but typically entails considerable fitness costs [27]–[30]. This is beneficial for resistance management because in the absence of carbamates or organophosphates, serine frequencies should fall rapidly [29], [31]. In *Culex pipiens*, duplications of *ACE-1* create linked serine and glycine alleles, which, when combined with an unduplicated serine allele, creates highly insecticide resistant genotypes with near-full wild-type functionality, thus providing a mechanism that can compensate for fitness costs [28], [31]. Worryingly, duplication has also been found in *An. gambiae* [23] though the consequences of copy number variation for fitness in the presence or absence of insecticide are not yet known in *Anopheles*. Though far from complete, information is available for metabolic resistance mechanisms to pyrethroids and DDT in wild populations of *An. gambiae* [5], [6], [32]–[34]. Indeed, a specific P450 enzyme, CYP6M2, has been demonstrated to metabolize both of these insecticide classes, suggesting the potential to cause cross-resistance in *An. gambiae* [32], [35]. By contrast little is known about metabolic mechanisms of carbamate resistance in mosquitoes and, as a consequence, potential for mechanisms of cross-resistance are unknown.

A particularly striking and potentially problematic example of insecticide resistance has been found in one of the two morphologically identical, but ecologically and genetically divergent molecular forms comprising the *An. gambiae s.s.* species pair (M molecular form, recently renamed as *An. coluzzii* [36]) in Tiassalé, southern Côte d'Ivoire. The Tiassalé population is resistant to all available insecticide classes, and displays extreme levels of resistance to pyrethroids and carbamates [11]. The *VGSC* 1014F (‘*kdr*’) and *ACE-1* G119S mutations are both found in Tiassalé [11], [25]. Yet *kdr* shows little association with pyrethroid resistance in adult females in this population [11]. *ACE-1* G119S is associated with both carbamate and organophosphate survivorship [11], but this mutation alone cannot fully explain the range of resistant phenotypes, suggesting that additional mechanisms must be involved. Here we apply whole genome microarrays, transgenic functional validation of candidates, insecticide synergist bioassays, target-site genotyping and copy number variant analysis to investigate the genetic basis of (1) extreme bendiocarb resistance and (2) cross-insecticide resistance in *An. gambiae* from Tiassalé. Our results indicate that bendiocarb resistance in Tiassalé is caused by a combination of target site gene mutation and duplication, and by specific P450 enzymes which produce resistance across other insecticide classes.

## Results

### Whole genome transcription analysis

**Figure 1:**
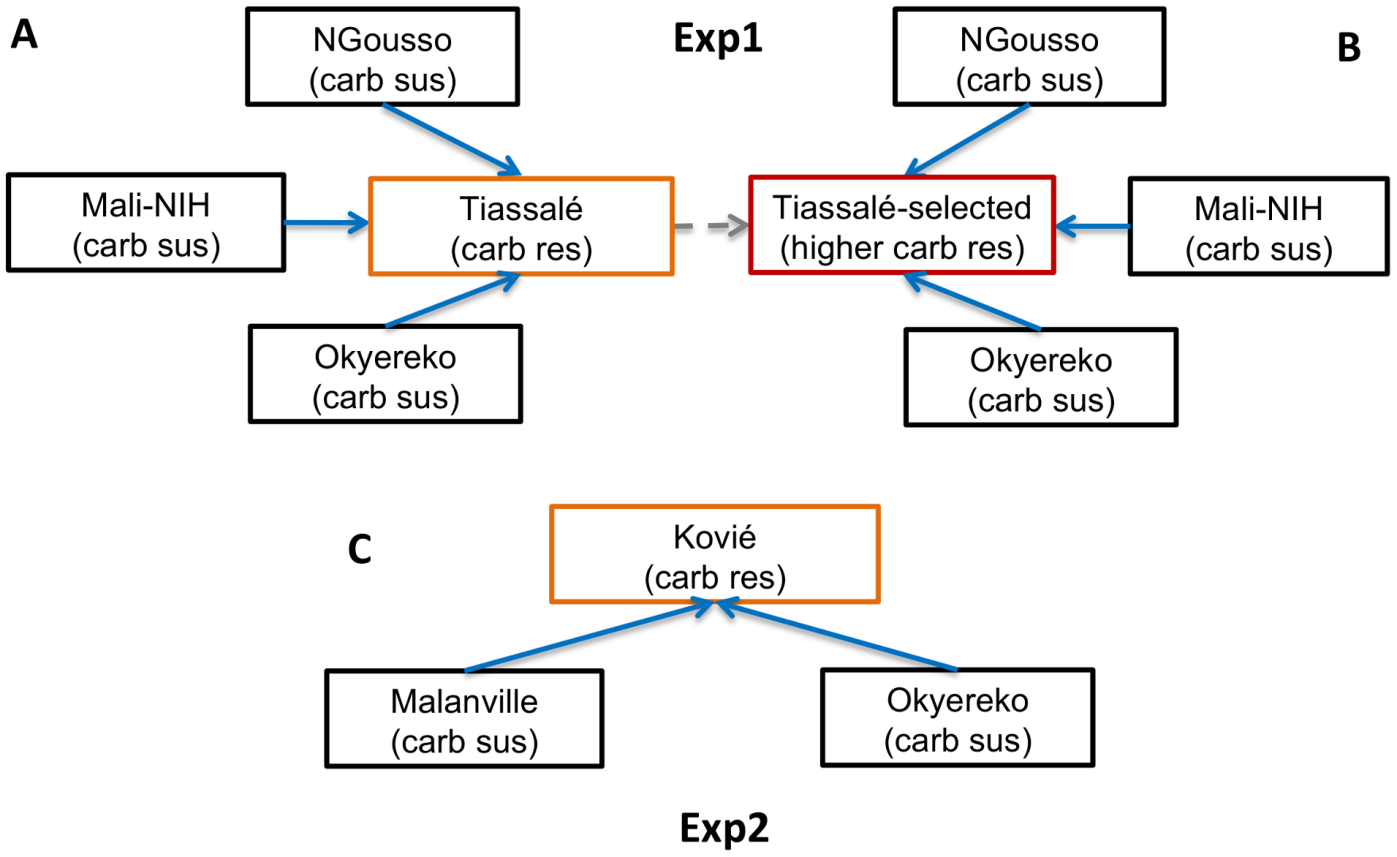
Microarray experimental design. Arrows indicate pairwise comparisons with direction indicating an increasing level of bendiocarb resistance, which was used to predict the expected direction of differential gene expression (only solid arrows were used to determine significance). Coloured boxes indicate samples resistant to bendiocarb; the red box indicates the only bendiocarb-selected sample. In Exp2 (C) microarray probes were considered significantly differentially expressed in resistant samples if: (i) each sus *vs.* res comparisons showed a consistent direction of expression as predicted by arrow direction; and (ii) each sus *vs.* res comparison yielded corrected P<0.05. In Exp1 (A, B) an additional criteria for significance was applied to increase specificity of results to the bendiocarb phenotype: (iii) fold-change for each Tiassalé-selected *vs.* sus comparison must be more extreme than the corresponding Tiassalé *vs.* sus comparison. Overall significance required significance in both Exp1 and Exp2.

Our study involved two microarray experiments (hereafter referred to as Exp1 and Exp2), involving solely M molecular form *An. gambiae* (Table S1), to identify candidate genes involved in bendiocarb resistance (full microarray results for Exp1 and Exp2 are given in Table S2A). In Exp1 gene expression profiles of female mosquitoes from bendiocarb-susceptible laboratory strains (NGousso and Mali-NIH) and a bendiocarb-susceptible field population (Okyereko, Ghana), none of which were exposed to insecticide, were compared to those of Tiassalé females. Two Tiassalé groups were used: either without insecticide exposure (Figure 1A), or the survivors of bendiocarb exposure selecting for the 20% most resistant females in the population [11] (Figure 1B). We used a stringent filtering process to determine significant differential expression (detailed in the legend to Figure 1), which included criteria on both the probability and consistency of direction of differential expression, and also required a more extreme level of differential expression in the Tiassalé-selected than Tiassalé (unexposed) *vs.* susceptible comparisons. Inclusion of this third criterion enhanced the likelihood that genes exhibiting differential expression are associated with bendiocarb resistance, rather than implicated via indirect association with another insecticide. Moreover, the requirement for significance in comparisons involving both bendiocarb-exposed and unexposed Tiassalé samples (Figure 1A, B) negates the possibility that any differential expression identified was a result solely of induction of gene expression by insecticide exposure.

**Figure 2:**
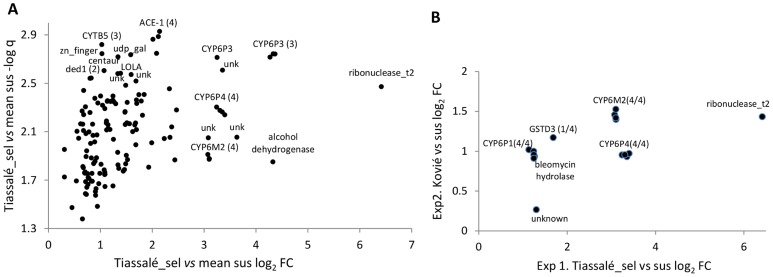
Genes significantly overexpressed (relative to susceptible samples) in (A) Tiassalé bendiocarb resistant samples in Exp1, and (B). both Tiassalé and Kovié samples. Plots show: (A). Log_2_-transformed fold-changes (FC) plotted against -log_10_ transformed q-values (multiple-testing-corrected probabilities) for bendiocarb-selected Tiassalé samples versus the average of the three susceptible populations; (B) Comparison of Kovié FC against Tiassalé-selected FC for probes significant in both experiments. For genes represented by multiple probes, numbers in parentheses indicate the number of probes significant/total.

In Exp1 145 probes were significant, out of a total of 14 914 non-control probes, with almost all (143/145) expressed at a higher level in the resistant samples (Table S2B). Functional annotation clustering analysis detected two significant clusters within the significantly over-expressed genes (Table S2C). The larger cluster was enriched for several P450s and the functionally-related genes cytochrome b5 and cytochrome P450 reductase. Of these, *CYP6P3*, *CYP6P4*, *CYP6M2* and cytochrome b5 are evident amongst the most significant and/or over-expressed probes in Figure 2A. Of the five physically-adjacent CYP6P subfamily genes in *An. gambiae*, *CYP6P1* and *CYP6P2* were also significant (Table S2B), and *CYP6P5* only marginally non-significant according to our strict criteria (five out of the six comparisons q<0.05). The four probes for the *ACE-1* target site gene exhibited the strongest statistical support (lowest q-values) for resistance-associated overexpression in the Exp1 dataset (Figure 2A).

Experiment 2 employed a simpler design in which bendiocarb resistant samples from Kovié (Togo) were compared to the same Okyereko field samples used in Exp1 and to a second field population from Malanville (Benin). Significant differential expression was determined according to the first two criteria employed for analysis of Exp1 (Figure 1). The likelihood of specificity of results to the bendiocarb resistance phenotype was enhanced because all three populations used in Exp2 exhibit resistance to pyrethroids and DDT, all are susceptible to organophosphates, but only the Kovié population is resistant to bendiocarb. In Exp2 2453 probes were significantly differentially expressed (Table S2D); likely reflecting the lower number of pairwise comparisons available for stringent filtering than in Exp1. Consequently we do not consider results from Exp2 alone in detail. Nevertheless it is interesting to note that the lowest q-values and highest fold-changes were both for alcohol dehydrogenase genes (Figure S1), and the latter is the physical neighbour and closest paralogue of the highly overexpressed alcohol dehydrogenase in Exp1 (Figure 2A). Sixteen probes, representing only seven genes, were significant in both Exp1 and Exp2 (Figure 2B), including all replicate probes for three of the CYP6 P450 genes highlighted previously. Of these, *CYP6M2* was most highly over-expressed, second only to *Ribonuclease t2*. However, results for *Ribonuclease t2* were much more variable, with differential expression dramatically high compared to lab strains, but moderate or low compared to wild populations (Table S2E). Evidence for specific involvement in bendiocarb resistance is suggested by significance of two of the *CYP6M2* probes in the (relatively low-powered) direct comparison of bendiocarb selected vs. unselected samples within Exp1; the other two *CYP6M2* probes and two of those for *ACE-1* were marginally non-significant (0.05<q<0.10; Figure S2).

### qRT-PCR expression of candidate genes

**Figure 3:**
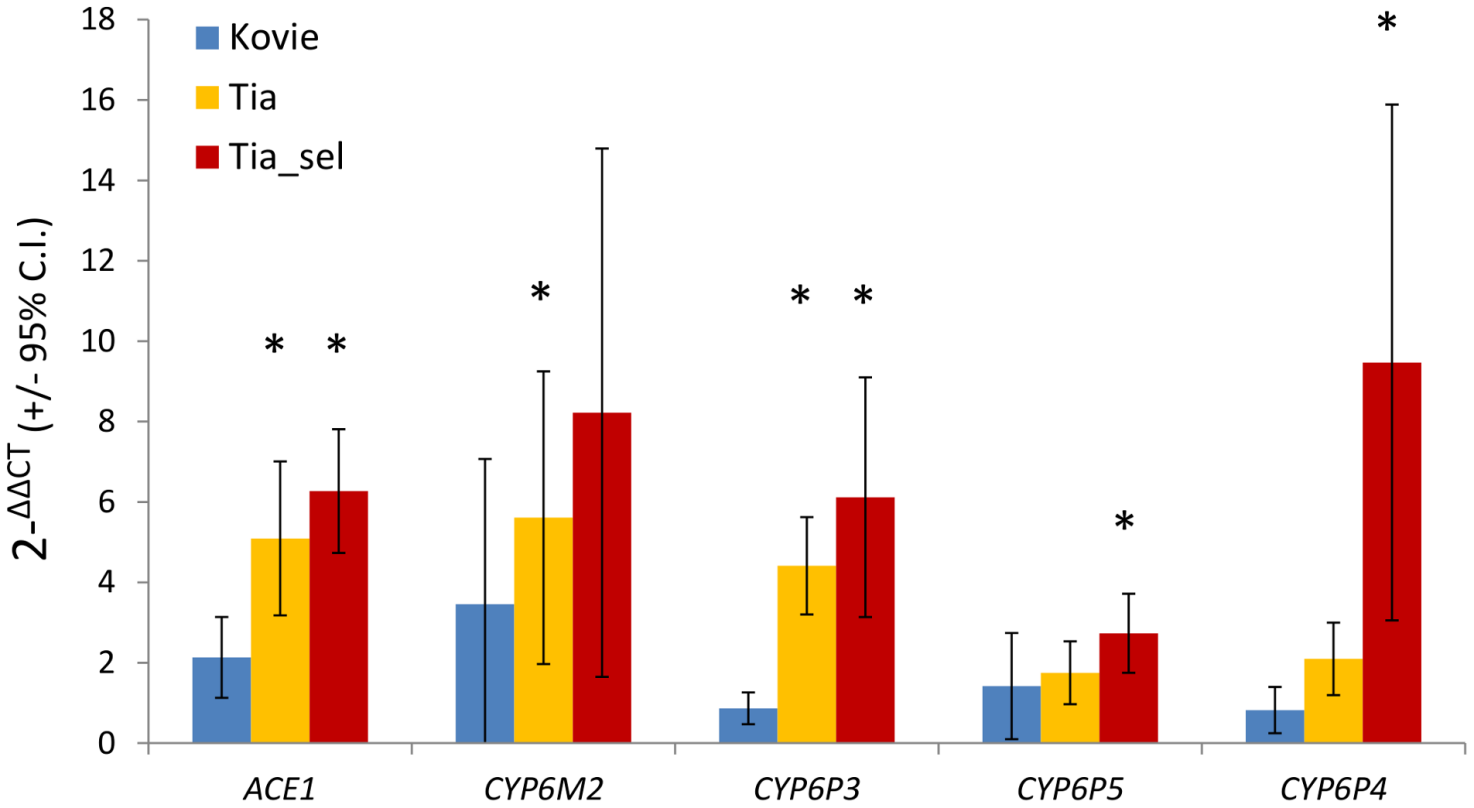
qRT-PCR expression analysis of candidate genes. Bars show mean fold changes relative to the bendiocarb and organophosphate susceptible Okyereko population. Asterisks indicate significant over-expression. Expression differences between pairs of populations are significant where error bars do not overlap. N = 5 biological replicates except for Tia_sel (N = 3).

Five genes were chosen for further analysis: ACE-1 and *CYP6P3* from Exp1; *CYP6M2* and *CYP6P4* from Exp1+Exp2; and *CYP6P5*, which we included because of a suspected type II error in the microarray analysis (see above). qRT-PCR estimates of expression, relative to the susceptible Okyereko population, showed reasonable agreement with microarray estimates albeit with some lower estimates (Figure S3). *CYP6M2* and *CYP6P4* exhibited up to eight and nine-fold overexpression, and *ACE-1* six-fold compared to Okyereko, though high variability among biological replicates for the P450 genes resulted in relatively few significant pairwise comparisons (Figure 3). Nevertheless the hypothesis that fold-changes should follow the rank order predicted by the level of bendiocarb resistance in each comparison (i.e. Tiassalé selected>Tiassalé unexposed>Kovié) was met qualitatively for all genes (Figure 3).

### Insecticide resistance phenotypes of CYP6 genes in *Drosophila*

**Figure 4:**
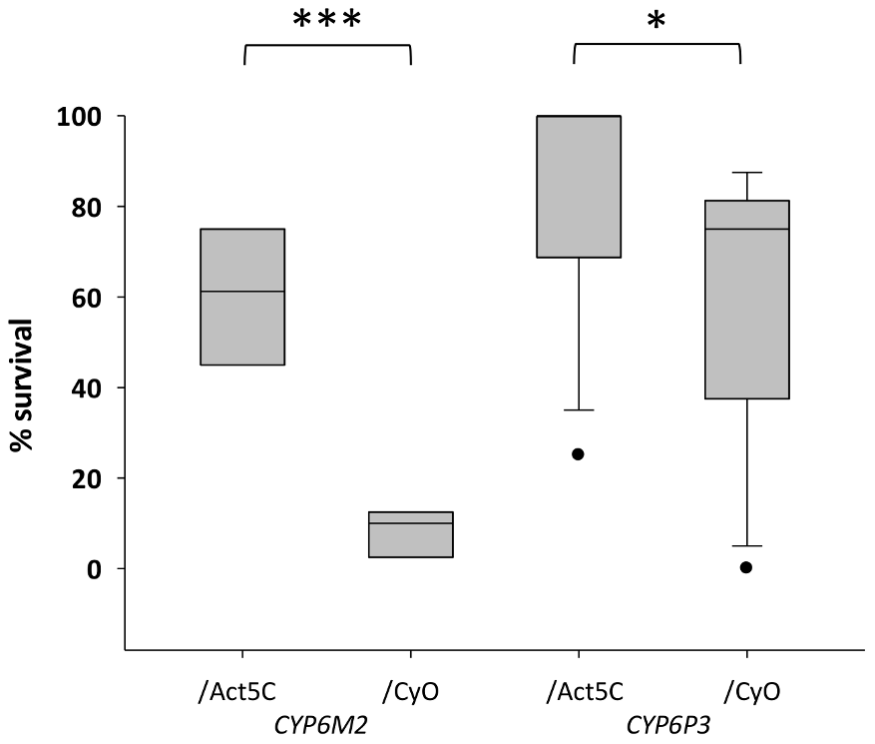
Survival of transgenic *Drosophila* expressing *An. gambiae Cyp6M2* or *CYP6P3* in the presence of bendiocarb. Boxes show interquartile ranges with median lines and whiskers (error bars) show 95^th^ percentiles for test (Act5C driver) or control (CyO) lines following exposure to 0.1 µg bendiocarb. Note that whiskers and median lines coincident with interquartile limits are not visible. Individual points falling outside percentiles are marked as dots. Mann-Whitney tests: ***P<0.001; *P<0.05.

For functional validation via transgenic expression in *D. melanogaster*, we chose *CYP6P3* and *CYP6M2*; both of which have been shown to metabolize pyrethroids [34], [35], and *CYP6M2* also DDT [32]. The capacity of each gene to confer resistance to bendiocarb, to the class I and II pyrethroids permethrin and deltamethrin, respectively, and to DDT and was assessed by comparing survival of transgenic *D. melanogaster*, exhibiting ubiquitous expression of *CYP6M2* or *CYP6P3* (e.g. UAS-*CYP6M2*/ACT5C-GAL4 experimental class flies), to that of flies carrying the *UAS-CYP6M2* or *CYP6P3* responder, but lacking the ACT5C-GAL4 driver (e.g. UAS-*CYP6M2*/CyO control class flies). For *CYP6M2* the relative expression level of the experimental flies was 4.0 and for *CYP6P3* 4.3 (Table S3). As indicated by elevated LC_50_ values (Figure S4), expression of either *CYP6M2* or *CYP6P3* produced pyrethroid resistant phenotypes, and *CYP6M2* expression also induced significant DDT resistance (Table 1). Assays for *CYP6P3* with DDT did not produce reproducible results (data not shown). Flies expressing the candidate genes exhibited greater survival across a narrow range of bendiocarb concentrations (Figure S4). However, at a discriminating dosage of 0.1 µg/vial [37] a resistance ratio of approximately seven was exhibited for *CYP6M2*/ACT5C: *CYP6M2*/CyO flies (Mann-Whitney, P = 0.0002; Figure 4) with a much smaller, but still significant, ratio of approximately 1.4 (Mann-Whitney, P = 0.019) for *CYP6P3*/ACT5C: *CYP6P3*/CyO flies. Caution is required in quantitative interpretation of the resistance levels generated, both because of the non-native genetic background and also ubiquitous expression of genes that may be expressed in a tissue-specific manner [38]. Nevertheless, the bioassays on transgenic *Drosophila* show that each P450s can confer resistance to more than one insecticide class.

**Table 1:** Survival of transformed *D. melanogaster* expressing *CYP6M2* and *CYP6P3* exposed to the pyrethroids permethrin and deltamethrin, and for *CYP6M2* also DDT.

	CYP6M2		CYP6P3	
	/Act5C	/Cy0	/Act5C	/Cy0
perm	**18.37**	4.97	**13.74**	5.56
	9.71–34.75	2.72–9.11	10.74–17.58	3.08–10.02
delta	**0.94**	0.11	**0.72**	0.09
	0.71–1.25	0.09–0.14	0.41–1.28	0.05–0.15
DDT	**4.04**	0.91		
	3.09–5.28	0.70–1.17		

LC_50_ estimates (µg) and 95% confidence limits are shown, in bold type where Act5C test line LC_50_s are significantly greater than CyO controls.

### *In vitro* metabolism assays

**Figure 5:**
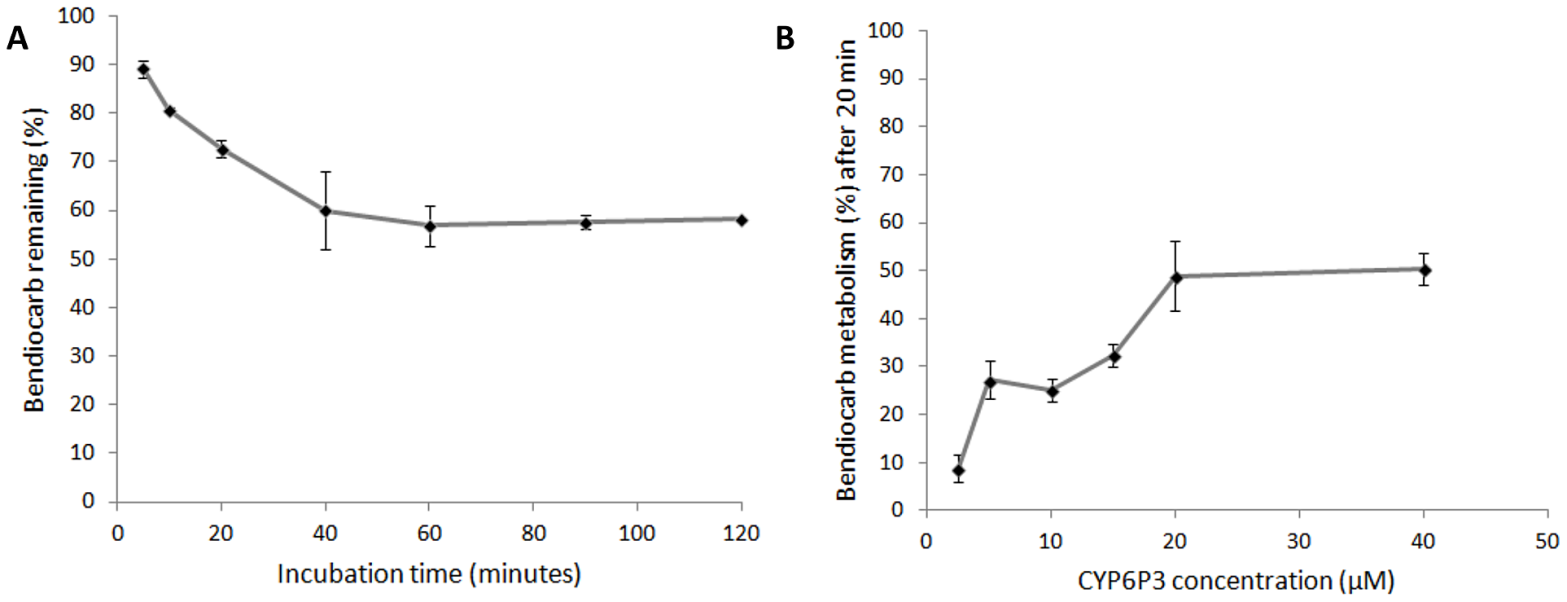
*In vitro* metabolism of bendiocarb by recombinant CYP6P3 expressed in *E. coli*. In both plots, which show the effect of (A) incubation time and (B) enzyme concentration, points show the mean of three replicates (following subtraction of no-NADPH negative control values) ± one standard error.

Recombinant CYP6M2 and CYP6P3 were expressed in E. coli with An. gambiae NADPH P450 reductase and cytochrome b5. An initial experiment, using 0.1 µM P450 and 2 hour incubation with bendiocarb, demonstrated metabolism of bendiocarb by CYP6P3 (64.2% mean depletion ±4.0% st.dev) but no metabolic activity of CYP6M2 (0±11.0%). Further investigation of CYP6P3 activity across a range of incubation times (Figure 5a) and enzyme concentrations (Figure 5b) supported the initial observation, with metabolism plateauing at a maximum of 50%.

### Resistance phenotypes and inhibition

**Figure 6:**
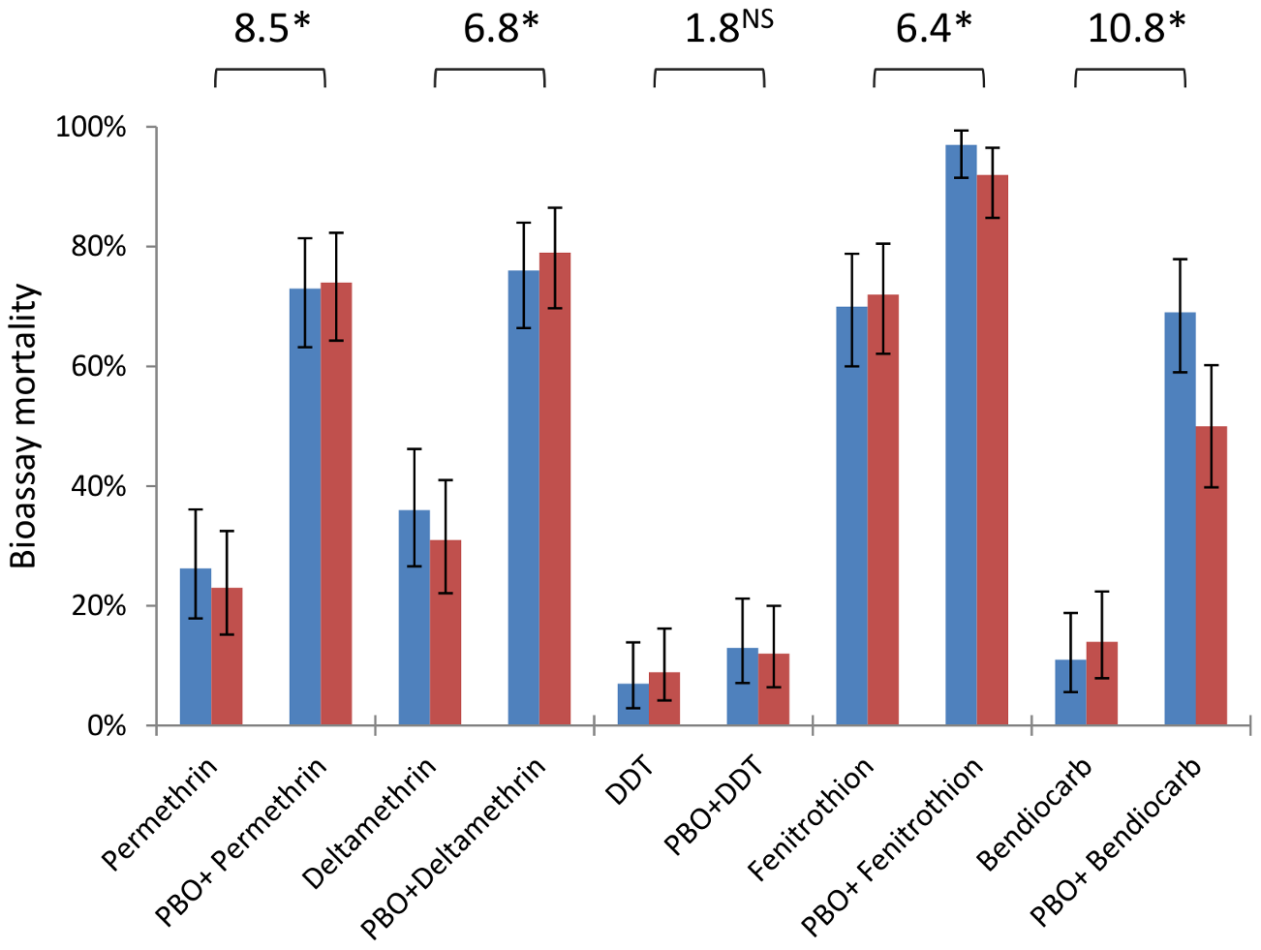
Insecticide resistance phenotypes from dry (blue) and wet (red) seasons with and without the synergist PBO. Bars are mean mortalities from four replicate bioassays (N = 25 each), with 95% binomial confidence limits. Odds ratios are shown above bars and represent the odds of mortality with PBO pre-exposure, compared to the odds of mortality with insecticide alone (data from the two seasons are pooled). *P≪0.001; ^NS^not significant (×^2^-test).

*An. gambiae* from Tiassalé are classified as resistant to all classes of WHO-approved insecticides (<90% bioassay mortality 24 hours after a 60 min exposure), with resistance phenotypes stable across wet and dry seasons (Figure 6, Table S4). Nevertheless, resistance varies markedly among insecticides (Table S4), with notably higher prevalence for bendiocarb and DDT than the organophosphate fenitrothion. The synergist PBO, which is primarily considered an inhibitor of P450 enzymes, exerted a significant influence on bioassay mortality (Table S4) for four of the five insecticides tested, with only DDT not significantly impacted (Figure 6). The synergising effect of PBO was strongest for bendiocarb, with a near five-fold increase in mortality, equivalent to an odds ratio for PBO-induced insecticidal mortality exceeding ten (Figure 6). However, for all of the insecticides, apart from fenitrothion, over 20% of the population survived even with PBO pre-exposure.

### AChE target site resistance

**Figure 7:**
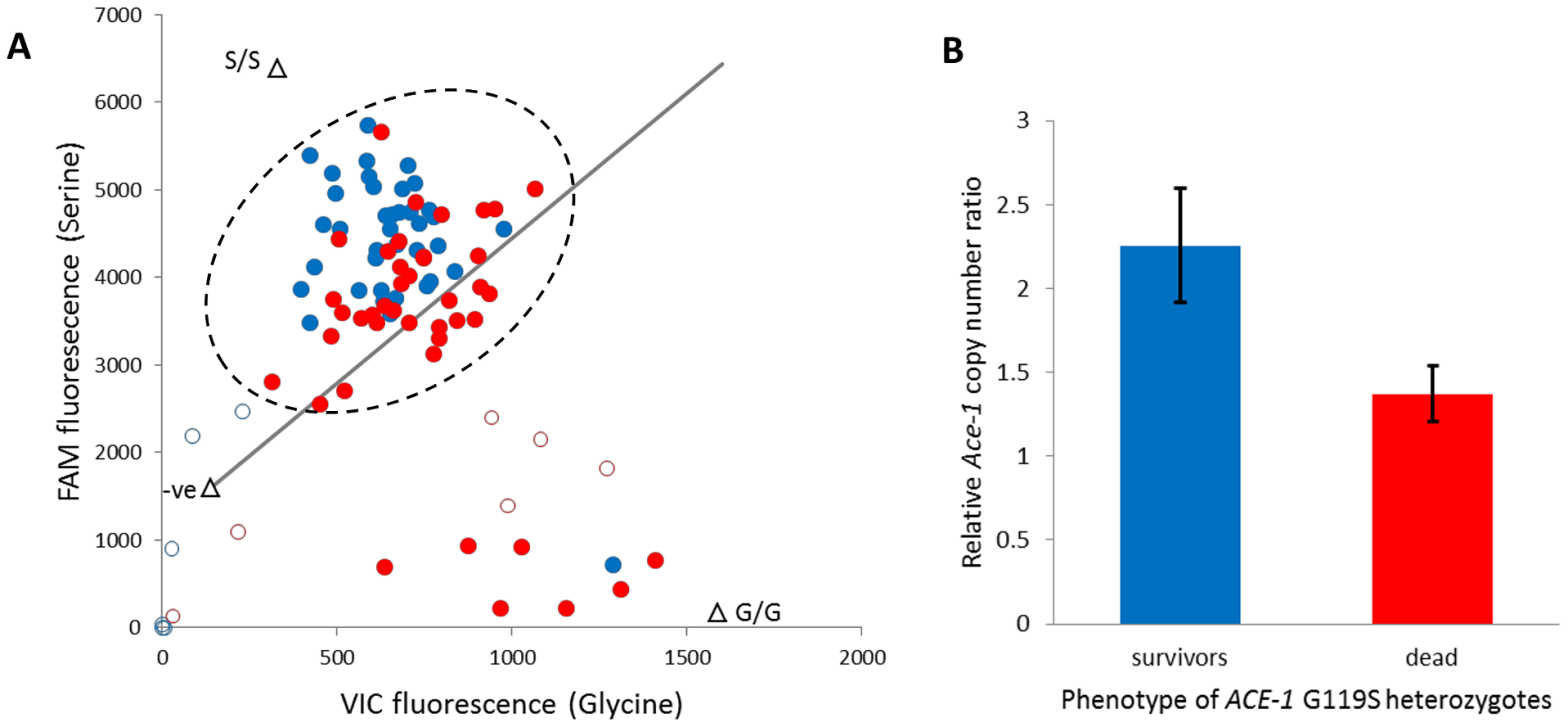
Role of target site allelic variation and copy number variation in bendiocarb resistance. A. *ACE-1* G119S TaqMan genotyping scatterplot of females exposed to bendiocarb, following PBO synergist exposure. Filled dots are genotypes called, unfilled are those excluded owing to ambiguous position. The line illustrates a 1∶1 Glycine (G): Serine (S) allele balance. Triangles are controls: S/S = mutant (resistant) allele homozygote; G/G = wild type (susceptible) allele homozygote. The line illustrates a 1∶1 Gly∶Ser allele balance. The dashed circle illustrates heterozygous genotypes. B. *Ace-1* genomic DNA copy number ratio of survivors and dead (N = 16 each) from the heterozygote genotype cluster. Bars show mean ΔΔ*CT* values relative to a standard susceptible laboratory strain (Kisumu) following normalisation against reference genes; error bars are 95% confidence intervals. In both plots blue denotes bioassay survivors and red denotes dead.

The *ACE-1* G119S substitution is the only non-synonymous target site mutation known in *An. gambiae* [23], and the resistant (serine) allele is common in Tiassalé with an estimated frequency of 0.46 (N = 306). All occurrences of serine are in heterozygotes (95% confidence limits for heterozygote frequency: 0.87–0.94), which underlies a dramatic deviation of genotype frequencies from Hardy-Weinberg equilibrium (÷^2^ = 135.5, P≈0). To examine the independence of putatively P450-mediated resistance and AChE target site insensitivity, we typed the G119S locus in females from the diagnostic (60 min) bendiocarb assays with and without pre-exposure to PBO. In either case absence of the 119 serine allele appears to almost guarantee mortality to bendiocarb (Table S5), as previously observed for fenitrothion bioassays in Tiassalé [11]. However, the strong bendiocarb resistance association of G119S was reduced significantly by PBO pre-exposure (homogeneity ÷^2^ = 8.3, P = 0.004) with the probability of survival for heterozygotes reduced to approximately 50% (Table S5). To investigate whether heterozygote survivorship might be linked to copy number variation, via a difference in numbers of serine and glycine alleles, we examined the qPCR dye balance ratio for live and dead individuals within the heterozygote genotype call cluster (Figure 7A). In many individuals called as heterozygotes, a markedly higher ratio of 119S: 119G dye label than the 1∶1 expected for a true heterozygote is evident (Figure 7A), and surviving heterozygotes exhibited a significantly higher serine: glycine dye signal ratio than those killed (t-test, P = 1.5×10^−5^). We designed an additional qRT-PCR diagnostic to investigate copy number more directly in a portion of the surviving and dead individuals typed as G119S heterozygotes. The difference in copy number was highly significant between survivors and dead (Figure 7B), with 15/16 survivors but only 5/16 dead females exhibiting a copy number ratio in excess of 1.5 (Table S5), consistent with possession of an additional allele. These results show that independent of the enzymes inhibited by PBO survival, females heterozygous for the G119S mutation (i.e. most individuals in Tiassalé) depends upon *Ace-1* copy number variation and possession of additional resistant serine alleles.

## Discussion

Bendiocarb is an increasingly important alternative to pyrethroids for IRS, but with carbamate resistant malaria vectors now established in West Africa [9]–[13] detailed understanding of the underlying mechanisms is urgently required to combat resistance and avoid cross-resistance [6]. Exhibiting the most extreme carbamate resistance and multiple insecticide resistance phenotypes documented to date in *An. gambiae* [11], the Tiassalé population represents an especially suitable model to address this question. Our results show how P450s contribute to multiple resistance in Tiassalé, and couple with overexpression of *ACE-1* resistant alleles to produce extreme bendiocarb resistance.

### P450s in carbamate resistance and cross-resistance

The major biochemical mechanisms of carbamate resistance in mosquitoes have previously been identified as modified AChE (via point substitutions, most notably G119S) and less frequently esterase-mediated metabolism [7]. PBO-induced increases in carbamate mortality have been reported in wild mosquito populations exhibiting low to moderate resistance levels, including M form *An. gambiae* from West Africa [12], [39],[40]. The significant synergizing effect of PBO in the present work and these previous studies is consistent with a role of P450s in carbamate resistance, but should not be taken alone as direct proof [41] because PBO exposure can also inhibit some esterases [42], [43]. However, our microarray data clearly identified over-expression of multiple *CYP6* P450 genes, whereas only a single carboxylesterase gene (*COEAE6G*) was significant, and expressed at a lower level (Table S2B). Taken together, the synergist data and transcriptional profiles indicate that a substantial proportion of the Tiassalé population is dependent upon the action of P450s for resistance to bendiocarb. Near-equivalent synergism of permethrin and deltamethrin, coupled with identification and functional validation of shared candidate genes, suggests the same conclusion for pyrethroids. For fenitrothion, the effect of PBO is also consistent with P450 involvement, but in the absence of specific candidate genes, additional supporting evidence will be required to confirm this hypothesis.

Genes from the CYP6P cluster emerged as strong candidates for involvement in P450-mediated detoxification. *CYP6P3* overexpression has been linked repeatedly with pyrethroid resistance in *An. gambiae* [33], [34], as has its orthologue in *An. funestus CYP6P9* [44], [45] and both enzymes can metabolise class I and II pyrethroids [34], [35], [45]. We demonstrate that *CYP6P3* can produce significant resistance to both classes of pyrethroid and, to a lesser extent bendiocarb, in *D. melanogaster*. We also show that recombinant *CYP6P3* can metabolise bendiocarb *in vitro*; the third mosquito P450 to metabolise a carbamates, after *An. gambiae* CYP6Z1 and CYP6Z2 which have been demonstrated to metabolise the insecticide carbaryl [46]. Interestingly *CYP6P4*, which, in contrast to *CYP6P3*, was also significantly overexpressed in the Togolese Kovié population, is the orthologue of the resistance-associated *CYP6P4* gene in *An. funestus* [44], and along with *CYP6P3* was recently found to be overexpressed in DDT-resistant samples of both M and S molecular forms of *An. gambiae* from Cameroon [47]. Although we were unable to obtain data for the impact of *CYP6P3* expression on survival with DDT exposure in *D. melanogaster*, the potential of CYP6P genes to act on DDT merits further investigation. It is also interesting to note that both cytochrome b5 and cytochrome P450 reductase, both important for P450-mediated insecticidal detoxification [48] are overexpressed in Tiassale, suggesting a possible role in resistance for co-expression of these genes with the CYP6 P450s.

*CYP6M2* was overexpressed in Tiassalé, Kovié, and also in the Tiassalé bendiocarb-selected *vs.* control comparison. *CYP6M2* expression generated *Drosophila* phenotypes significantly resistant to bendiocarb, DDT, and class I and II pyrethroids. Overexpression of *CYP6M2* has been linked repeatedly to pyrethroid [33], [34] and DDT resistance [32], [47] in *An. gambiae*, and is known to metabolise both these classes of insecticide [32], [35]. Our data now suggest a role in bendiocarb resistance, and overall provide strong evidence for involvement in resistance to three classes of insecticide. The biochemical mechanism of involvement remains unclear however because CYP6M2 did not metabolise bendiocarb *in vitro*, though we cannot rule out the possibility that some unknown, and thus currently, absent co-factor might be required. Sequestration also seems unlikely since CYP6M2 does not appear to bind bendiocarb. A role in breakdown of secondary bendiocarb metabolites certainly remains plausible, though at present knowledge of such mechanisms for any insecticide in mosquitoes is very limited [49], [50]. High variability in *CYP6M2* expression among biological replicates, especially evident in qRT-PCR, suggests that the regulatory mechanism(s) generating overexpression is far from fixation in Tiassalé. Further work is required to determine whether the cause of overexpression might be gene amplification, as seen for insecticide-linked CYP6P genes in An. funestus [44] and *CYP6Y3* in the aphid *Myzus persicae* [51] or a *cis* regulatory variant, or both, as documented for *CYP6G1* in *D. melanogaster* [52]. In either case, the actual level of expression in individuals possessing causal regulatory variant(s) may be much higher than we detected from pooled biological replicates. As a consequence, it is possible that *CYP6M2* (and other key P450s) might be expressed at too high a level for PBO to fully inhibit at the dosage applied, resulting in only partial synergy. Indeed it is interesting that *CYP6M2* generated significant DDT resistance in transformed *Drosophila* in our study and has been shown metabolise DDT [32] yet PBO provided only very slight and non-significant synergy for DDT-exposed Tiassalé females. An inadequate concentration of PBO might be important, but it is worth noting that levels of DDT resistance in West African *An. gambiae* can be extreme and are likely to be underpinned by additional mechanisms [32] such as the significantly resistance-associated *kdr* L1014F target site mutation in Tiassalé [11]. Whilst incomplete synergy of highly expressed P450 enzymes might be a partial explanation, our results point to target site mechanisms as a key factor underpinning survival following PBO and bendiocarb exposure.

### Target site insensitivity and amplification

Possession of the *ACE-1* 119 serine variant appears to be a near-prerequisite for bendiocarb-survival in Tiassalé, as documented previously for fenitrothion [11]. This is apparently not the case in all *An. gambiae* populations, with some individuals lacking the serine mutation surviving a standard 60 min exposure [12], [39]. Over 90% of Tiassalé mosquitoes are heterozygous for G119S, which could be consistent with fitness costs for individuals lacking a fully-functional wild-type allele since the serine allele exhibits lowered activity [28]. It is apparent though that possession of the *ACE-1* G119S mutation represents only a portion of the target site mediated resistance mechanism. Tiassalé females generally showed much higher expression of *ACE-1* than all other populations in our experiments, reaching approximately six-fold in the highly resistant bendiocarb-selected group compared to the Okyereko susceptible group. Following PBO-mediated P450 inhibition, survival of G119S heterozygotes was reduced to approximately 50% and our results show that individuals exhibiting a higher *ACE-1* copy number and more copies of the serine allele had a significant survival advantage. Together these results indicate that the primary explanation for the ubiquitous heterozygosity found in Tiassalé is an elevated copy number of expressed *ACE-1* alleles. At least in individuals possessing additional serine alleles, this enhances carbamate resistance, and can apparently generate resistance independently of P450 activity.

Extra copies of *ACE-1* alleles have been found in West African *An. gambiae*, and lack of sequence variation suggests that duplication is a very recent event [23]. Consequences of *ACE-1* duplication have not been documented previously in *Anopheles* but *Cx. pipiens* possessing two G119S resistant alleles and a wild type susceptible allele can exhibit near maximal fitness in the presence and absence of organophosphate treatment [30]. If this fitness scenario is similar in *An. gambiae ACE-1* duplicates could spread rapidly, or may have already done so but have been largely undetected by available diagnostics. The estimated copy numbers we detected in some individuals suggests that more *ACE-1* copies may be present in *An. gambiae* than are known in *Cx. pipiens*, perhaps more akin to the high level of amplification found in spider mites *Tetranychus evansi* [53]. This raises the possibility of a potentially multifarious set of resistant phenotypes dependent upon the number and G119S genotype of the copies possessed by an individual, understanding of which will benefit from further application of the DNA-based qPCR diagnostic we have developed.

### Conclusion

Extreme levels of resistance to single insecticides, and multiple resistance across different insecticidal classes represent major problems for control of disease vectors, and pest insects generally. Tiassalé *An. gambiae* show exceptionally high-level carbamate resistance and the broadest insecticide resistance profile documented to date. Our results indicate that overexpression of specific CYP6 enzymes and duplicated resistant *ACE-1* alleles are major factors contributing to this resistance profile. Results from the less resistant Kovié population show that at least some of the mechanisms are not restricted to Tiassalé and could be quite widespread in West Africa. The involvement of *CYP6P3* and *CYP6M2* in resistance to multiple insecticide classes parallels the cross resistance engendered by *CYP6* genes in other insect taxa [54], [55] and is extremely concerning because resilience to standard resistance management strategies is likely to be increased greatly. Further work is now required to understand the biochemical role of CYP6M2 in detoxification of bendiocarb and also to better understand any associated fitness costs of elevated CYP6P gene expression. In addition, whilst we have demonstrated involvement of elevated expression of the CYP6 P450s in insecticide resistance, the impact of structural variants within these genes remains to be investigated and is very poorly understood for P450-mediated insecticide resistance in mosquitoes. In spite of a major impact of PBO on three distinct insecticide classes, too many females remained alive to suggest that PBO provides a resistance-breaking solution. Nevertheless, we suggest that this preliminary conclusion may be worth further testing: (i) using higher PBO concentrations; (ii) in females old enough to transmit malaria, which are usually less insecticide resistant [56]–[58]; or (iii) in less resistant populations. Monitoring the spread of *ACE-1* duplications should be an immediate priority, whereas modification of AChE-targeting insecticides to reduce sensitivity to the G119S substitution [59], [60] represents an important longer-term goal.

## Materials and Methods

### Study design and samples

Our study involved *Anopheles gambiae* samples for bioassays coupled with target site genotyping and copy number analysis, and two microarray experiments. The first (Exp1; see Figure 1A, B) compared samples from laboratory strains or field populations entirely susceptible to carbamates, with bendiocarb-resistant females from Tiassalé, which were also the subject of bioassays. Exp2 (see Figure 1C) involved a comparison of a population moderately resistant to bendiocarb (Kovié) with two fully carbamate susceptible field populations. Sample site details and resistance profiles for each population or strain used in the microarrays are given in Table S1. For field populations, larvae were collected and provided with ground TetraMin fish food. Emerged adults were provided 10% sugar solution. All 3–5 day old females for subsequent gene expression analysis were preserved in RNALater (Sigma). With the exception of a selected group from the Tiassalé population (below), all samples were preserved without exposure to insecticide. The Tiassalé selected group were survivors of exposure to 0.1% bendiocarb (using WHO tubes and papers) for 360 min which induces approximately 80% mortality after 24 h (11); unexposed controls were held for 360 min with control paper, which did not induce mortality. All mosquitoes used in the study were identified as *An. gambiae* s.s. M molecular form using the SINE-PCR method [61].

### Synergist bioassays, *ACE-1* G119S genotyping and copy number analysis

The effect of the insecticide synergist piperonyl butoxide (PBO), a primary action of which is to inhibit P450 monooxygenase enzymes [41], was evaluated using WHO bioassays. Eight replicates of 25 adult female *An. gambiae* emerging from larvae obtained from an irrigated rice field in Tiassalé were exposed to five insecticides (permethrin, deltamethrin, DDT, bendiocarb and fenitrothion). Immediately prior to each 60 min insecticide exposure, mosquitoes were exposed to 4% PBO paper for 60 min. 100 females were exposed to PBO alone as control. Chi-squared tests were used to compare the mortality with and without PBO. A TaqMan qPCR assay [62] run on an Agilent Stratagene real-time thermal cycler was used to genotype PBO-exposed samples for the *ACE-1* G119S polymorphism, with qualitative calling of genotypes based on clustering in endpoint scatterplots. G119S genotype call data for samples not exposed to PBO was taken from a prior publication [11]. Following qualitative genotype calling, endpoint dR values for each dye were exported, and the data from individuals called as heterozygotes was analyzed quantitatively to investigate the possibility of sub-grouping within this genotype cluster. Specifically we tested whether surviving and dead mosquitoes, heterozygous for G119S, might possess different numbers of serine and alleles by comparing FAM (serine label)/VIC (glycine label) dye ratios using an unequal variance t-test. To further quantify the copy number variation suggested by the TaqMan genotyping results we designed a qRT-PCR to amplify fragments from three different exons of the *ACE-1* gene, with normalisation (for varying gDNA concentration among samples) provided via comparison with amplification of a fragment from each of two single-copy genes *CYP4G16* and *Elongation Factor*. Primer details are given in Table S6 and qRT-PCR conditions are the same as listed below for gene expression analysis. Relative copy number levels for *Ace-1* were estimated relative to two pools of samples (N = 4 each) from the Kisumu laboratory strain by the ΔΔCT method [63]. ΔΔCT values for each test sample are the mean for the three *ACE-1* amplicons following normalisation to both single copy genes and subtraction of the average normalised Kisumu values. Test samples were 16 *ACE-1* G119S heterozygote survivors and 16 dead, chosen at random from those genotyped by the TaqMan assay. ΔΔCT values were compared between survivors and dead using an unequal variance t-test.

### Microarrays

Total RNA was extracted from batches of 10 mosquitoes using the Ambion RNAqueous-4PCR Kit. RNA quantity and quality was assessed using a NanoDrop spectrophotometer (Thermo Fisher Scientific) and a 2100 Bioanalyzer (Agilent Technologies) before further use. Three biological replicate extractions of total RNA from batches of 10 mosquitoes for each sample population or colony (except Ngousso where there were N = 2 replicates) were labelled and hybridised to *Anopheles gambiae* 8×15 k whole genome microarrays using previously described protocols [32]. Exp 2 employed a fully-interwoven loop design (Figure S6), optimal for study power [64] whilst, owing to the large number of comparisons and unbalanced replication, a pairwise full dye-swap design was used for Exp1 with indirect connection through the (resistant) Tiassalé groups (Fig. 1 A, B). Exp1 was analysed using GeneSpring GX v9.0 software (Agilent), which is readily applied to dye swap experiments, while the R program MAANOVA [65], with LIMMA [66] for normalisation prior to ANOVA, was used to analyse the interwoven loop in Exp2, using previously-described custom R-scripts [32]. For both experiments, the basic significance threshold for any single pairwise comparison was a q-value with false discovery rate (FDR) set at 0.05 (i.e. an FDR-corrected threshold for multiple testing). Full details of the criteria applied to determine overall significance within and across Exp1 and 2 are given in Figure 1. Within Exp1, the direct comparison of Tiassalé bendiocarb-selected *vs.* Tiassalé control comparison was analysed separately and not used to determine overall significance, owing to the lower power expected for a within-population experiment involving the same level of replication as the cross-population comparisons [34]. Significantly over-expressed genes emerging from Exp1 were studied at functional level using the software DAVID Bioinformatics resources 6.7 [67]. Microarray data are deposited with ArrayExpress under accession numbers E-MTAB-1903 (Exp1) and E-MTAB-1889 (Exp2).

### qRT-PCR

Quantitative real-time PCR was used to provide technical replication of results from the microarray experiments for a subset of significantly over-expressed genes. Samples were converted to cDNA using oligo(dT)_20_ (Invitrogen) and Superscript III (Invitrogen) according to the manufacturer's instructions and purified with the QIAquick PCR Purification Kit. Three pairs of exon-spanning primers were designed for each gene of interest and from each triplicate a pair was chosen that produced a single peak from melt cure analysis, and PCR efficiency closest to 100%, determined using a cDNA dilution series obtained from a single sample. Primers details are listed in Table S7. All qRT-PCR reactions were run on an Agilent Stratagene real-time thermal cycler and analysed using Agilent's MXPro software (Mx3005P). The PCR conditions used throughout were 10 min for 95°C, 40 cycles of 10 s at 95°C and 60°C respectively, with melting curves run after each end point amplification at 1 min for 95°C, followed by 30 s increments of 1°C from 55°C to 95°C. The same RNA samples used for microarrays from Tiassalé (selected and unexposed), Kovié and Okyereko plus an additional two replicates (N = 5 for all but the Tiassalé selected group where N = 3) were used. Expression levels for each gene of interest were estimated relative to the Okyereko population (chosen as the reference bendiocarb susceptible group because it was present in both microarray experiments) by the ΔΔCT method following correction for variable PCR efficiency [63], and normalisation using two stably-expressed genes (*Rsp7* and *Elongation Factor*); primers and efficiencies are listed in Table S7. Statistical significance of over-expression of each group relative to Okyereko was assessed using equal or unequal variance t-tests as appropriate, depending on results of F-tests for homoscedasticity.

### Production of transgenic *Drosophila melanogaster*

cDNA clones containing the open reading frames for *CYP6M2* and *CYP6P3* (sequences from the *An. gambiae* Kisumu laboratory strain) were PCR-amplified using high fidelity AccuPrime Pfx polymerase (Invitrogen). PCR primers contained EcoRI and NotI restriction sites within the forward and reverse primers, respectively. PCR products were gel-purified using the GenElute Gel Extraction Kit (Sigma) and subsequently digested with the aforementioned restriction enzymes (New England Biolabs). The pUAST-attB plasmid (obtained from Dr. Konrad Basler, University of Zurich) digested with *Eco*RI and *Not*I was gel purified, as noted above, and incubated with PCR-amplified, restriction enzyme-digested products of the *CYP6M2* or *CYP6P3* clone and T4 DNA ligase (New England Biolabs). Ligation mixtures were transformed into competent DH5α cells, and individual colonies were verified using PCR. The EndoFree Plasmid Maxi Kit (Qiagen) was utilized to obtain large amounts of plasmids for subsequent steps. pUAST-attB clones containing the *CYP6M2* or *CYP6P3* insertion were sent to Rainbow Transgenic Flies, Inc. (Camarillo, CA, USA) for injection into Bloomington Stock #9750 (y^1^ w^1118^; PBac{y^+^-attP-3B}VK00033) embryos. The PhiC31 integration system in this stock enables site-specific recombination between the integration vector (pUAST-attB) and a landing platform in the fly stock (attP) [68]. Upon receiving the injected embryos, survivors were kept at 25°C, and G_o_ flies that eclosed were sorted by sex prior to mating. To establish families of homozygous transgenic flies, G_o_ flies were crossed with w^1118^ flies, and G_1_ flies were sorted based on w^+^ eye color (as a marker for insertion events). G*_1_* w^+^ flies were crossed *inter se* to obtain homozygous insertion lines. The following *D. melanogaster* stocks were obtained from the Bloomington *Drosophila* Stock Center (Bloomington, IN, USA): y^1^ w^1^; P{Act5C-GAL4}25FO1/CyO, y^+^, w^*^ (BL4414); P{GawB}Aph-4c232 (BL30828), and w^1118^ (BL3605). Virgin females from *CYP6M2* or *CYP6P3* insertion stocks were crossed with Act5C-GAL4/CyO (ubiquitous Actin5C driver) flies for expression studies.

### Transcript expression analysis

For each class within a cross (control and experimental), 8–10 two-day-old flies were obtained and flash-frozen in liquid nitrogen, and then stored at −80°C in triplicate. Total RNA was extracted using TRI Reagent (Sigma), and 1 µg of RNA was treated with RNase-Free DNaseI (Fisher Scientific). For each synthesis, a 10 µL reaction was created using 1 µL DNase-treated RNA; three technical replicates were performed for each biological replicate. Primers for amplification of cDNA product, used at a concentration of 0.75 µM, were: Cyp6M2_Forward: 5′-ACGAGTTCGAGCTGAAGGAT-3′, Cyp6M2_Reverse: 5′-GTTACACTCAATGCCGAACG-3′, Cyp6P3_Forward: 5′-TATTGCAGAGAACGGTGGAG-3′, Cyp6P3_Reverse: 5′TACTTCCGAAGGGTTTCGTC-3′. Relative expression was compared using Actin primers [69] at a concentration of 0.50 µM. qRT-PCR reactions were performed using USB VeriQuest SYBR Green One-Step qRT-PCR Master Mix (2X) on a 7500 Fast Real-Time PCR System (Applied Biosystems). Cycling conditions used were 50°C for 10 minutes and 95°C for 10 minutes, followed by 40 cycles of 90°C for 15 seconds and 56°C for 30 seconds, with the fluorescence measured at the end of each cycle.

### Bendiocarb metabolism assays

Recombinant CYP6M2 and CYP6P3 were commercially co-expressed with *An. gambiae* NADPH P450 reductase and cytochrome b5 in an E. coli system by Cypex (Dundee, UK). Using previously described methodologies [35] a first experiment showed that CYP6M2 was unable to metabolise bendiocarb (10 µM) after a 2 hour incubation and thus only CYP6P3 was investigated in subsequent experiments. For time course measurements, reactions were performed in 200 µL with 10 µM insecticide, 0.1 µM CYP6P3 membrane in 200 mM Tris-HCl pH 7.4 and started by adding the NADPH regenerating system (1 mM glucose-6-phosphate (G6P), 0.25 mM MgCl_2_, 0.1 mM NADP^+^, and 1 U/mL glucose-6-phosphate dehydrogenase (G6PDH)). Reactions were incubated for a specified time at 30°C with 1200 rpm orbital shaking and stopped by adding 0.2 mL of acetonitrile. Shaking was carried for an additional 10 min before centrifuging the reactions at 20000 *g* for 20 min. 200 µl of supernatant was used for HPLC analysis. Reactions were performed in triplicate and compared against a negative control with no NADPH regenerating system to calculate substrate depletion. An additional experiment with different enzyme concentrations was performed, using the methods above, for 20 mins with P450 concentrations of: 0.2, 0.1, 0.075, 0.05, 0.025 and 0.0125 µM. The reactions were performed in parallel against a negative control (−NADPH).

In each experiment the supernatants were analyzed by reverse-phase HPLC with a 250 mm C18 column (Acclaim 120, Dionex) and a mobile phase consisting of 35% acetonitrile and 65% water. The system was run at a controlled temperature of 42°C with 1 ml/min flow rate. Bendiocarb insecticide was monitored at 205 nm and quantified by measuring peak areas using OpenLab CDS (Agilent Technologies). Retention time was around 14.9 minutes.

### Insecticide exposure assays

An appropriate amount of insecticide was added to 100 µl of acetone and placed into individual 16×200 mm glass disposable culture tubes (VWR Scientific). Tubes were then placed on their sides and rotated continuously, coating the entire interior of the tube, until all acetone was evaporated. A total of 8–12 control and 8–12 experimental transgenic flies, aged 3–5 days post-eclosion, were added to each tube. Flies from experimental and control classes were mixed in single insecticide-coated vials for assays, to ensure equivalent exposure to insecticide. The tubes were capped with cotton balls saturated with a 10% (w/v) glucose/water solution. Tubes were then incubated at 25°C for 24 h, after which mortality was assessed. Linear regression models were used to fit dose-response curves, from which LC50 values (and confidence intervals) were estimated using Prism v5.0. However, for bendiocarb this was not possible owing to a very sharp inflection in the dose-response profile. Instead differences between lines were assessed at a diagnostic dose of 0.1 µg bendiocarb/vial, applied previously to *Apis mellifera* [37], [70], using Mann-Whitney U tests.

## Supporting Information

Figure S1 Probes significantly over-expressed in Kovié, relative to Okyereko and Malanville (Exp2). Average log_2_-transformed fold- changes are plotted against average -log_10_-transformed q-values (multiple-testing-corrected probabilities). An arbitrary cut-off of log_2_FC = 2 and –log q = 3 was used to determine probes to be labelled.
(PPTX)

Figure S2 Microarray results for Tiassalé selected vs unexposed controls. Arbitrary cut-offs of log2FC = 0.6 and –log q = 1 are used to determine points to label. (n) indicates label represents >1 replicate probes.
(PPTX)

Figure S3 Relationship between expression measured by qRT-PCR and microarrays for candidate genes. The overall correlation is r = 0.50 (P = 0.056).
(PPTX)

Figure S4 Survival of transgenic *D. melanogaster* that express *CYP6M2* or *CYP6P3* in the presence of varied amounts of insecticides. Log-linear plots of insecticide concentration vs. survival are shown. Blue points show survival of transformed flies with the *Act5C* driver which exhibit ubiquitous expression; red points show *CyO* control class flies. Bars show SEM of percent survival. Owing to the sharp inflection for both bendiocarb plots the regression model could not be applied to either *Act5C* or *CyO* data. N = 5 for all insecticides and concentrations other than bendiocarb at 0.1 µg, for which N = 8 (see Fig. 4). The gap in the x-axis results from use of a log scale on which control vials (zero insecticide) have no value.
(PPTX)

Figure S5 Distributions of *Ace-1* copy number ratios. Estimates are calculated relative to the susceptible Kisumu strain in Tiassalé samples genotyped as G119S heterozygotes that survived or died in a bendiocarb bioassay following PBO pre-exposure.
(PPTX)

Figure S6 Interwoven microarray experimental loop design used in Exp2 comparing field samples from Kovie (KOV) with Malanville (MAL) and Okyereko (OKY) Each pool, indicated by a circle, represents mRNA extracted from 10 female *An. gambiae* s.s. M form mosquitoes. Arrows indicate individual microarrays (N = 18 in total), with direction representing microarray cy dye labelling.
(PPTX)

Table S1 Details of *An. gambiae* samples used in experiments 1 and 2 (.xlsx).
(XLSX)

Table S2 Microarray results (.xlsx). **Table S2A**. Full microarray results from both experiments. **Table S2B**. Significant microarray probes from Exp1. **Table S2C**. DAVID functional annotation clustering for Exp1. **Table S2D**. Significantly microarray probes from Exp2. **Table S2E**. Microarray probes significant in both Exp1 & Exp2.
(XLSX)

Table S3 qRT-PCR expression results for transformed *Drosophila melanogaster*.
(DOCX)

Table S4 GLM testing factors effecting bioassay mortality.
(DOCX)

Table S5 Resistance association of the G119S target site mutation.
(DOCX)

Table S6 qRT-PCR primer details for copy number variant analysis.
(XLSX)

Table S7 qRT-PCR primer details for gene expression analysis.
(XLSX)
