# Supplementary material for: CYP6 P450 Enzymes and ACE-1 Duplication Produce Extreme and Multiple Insecticide Resistance in the Malaria Mosquito Anopheles gambiae
Source: PLoS Genet. 2014 Mar 20;10(3):e1004236. doi: 10.1371/journal.pgen.1004236 (PMC3961184; doi:10.1371/journal.pgen.1004236)
Supplement: Table S3 — qRT-PCR expression results for transformed Drosophila melanogaster. (DOCX) [file pgen.1004236.s009.docx]

**Table S3**. qRT-PCR expression results for transformed *Drosophila melanogaster*. Relative fold differences in expression between experimental and control flies (ΔΔCT) are highlighted for each gene. Biological replicates are in rows and technical replicates in columns.

| ***CYP6M2*** |  | CYP6M2/Act5C-Actin Primers | | | | CYP6M2 Primers | | |  |  | Experimental |
| --- | --- | --- | --- | --- | --- | --- | --- | --- | --- | --- | --- |
| Replicate | Tech 1 | Tech 2 | Tech 3 | mean |  | Tech 1 | | Tech 2 | Tech 3 | mean | ΔCT |
| 1 | 24.19 | 24.62 | 24.71 | 24.51 |  | 26.57 | | 26.68 | 26.61 | 26.62 | -2.11 |
| 2 | 20.24 | 21.43 | 21.79 | 21.15 |  | 27.31 | | 27.29 | 27.20 | 27.27 | -6.11 |
| 3 | 20.94 | 21.97 | 22.70 | 21.87 |  | 27.63 | | 27.56 | 27.51 | 27.57 | -5.70 |
|  |  |  |  |  |  |  | |  |  | mean | -4.64 |
|  |  | CYP6M2/CyO-Actin Primers | | | | CYP6M2 Primers | | |  |  | Control |
| Replicate | Tech 1 | Tech 2 | Tech 3 | mean |  | Tech 1 | Tech 2 | | Tech 3 | mean | ΔCT |
| 1 | 18.97 | 21.18 | 21.05 | 20.40 |  | 27.04 | 26.92 | | 27.07 | 27.01 | -6.61 |
| 2 | 21.48 | 21.94 | 22.49 | 21.97 |  | 29.40 | 29.62 | | 29.73 | 29.58 | -7.61 |
| 3 | 24.35 | 24.15 | 23.41 | 23.97 |  | 29.56 | 29.54 | | 29.90 | 29.67 | -5.70 |
|  |  |  |  |  |  |  |  | |  | mean | -6.64 |
|  |  |  |  |  |  |  |  | |  | **ΔΔCT** | **4.00** |
| ***CYP6P3*** |  | CYP6P3/Act5C-Actin Primers | | | | CYP6P3 Primers | | |  |  | Experimental |
| Replicate | Tech 1 | Tech 2 | Tech 3 | mean |  | Tech 1 | Tech 2 | | Tech 3 | mean | ΔCT |
| 1 | n/a | 26.10 | 26.51 | 26.31 |  | 24.20 | 24.06 | | 24.26 | 24.17 | 2.13 |
| 2 | 22.80 | 22.98 | 23.88 | 23.22 |  | 24.55 | 24.81 | | 24.67 | 24.68 | -1.46 |
| 3 | 17.24 | 18.25 | 18.23 | 17.91 |  | 24.93 | 25.48 | | 24.65 | 25.02 | -7.11 |
|  |  |  |  |  |  |  |  | |  | mean | -2.15 |
|  |  | CYP6P3/CyO-Actin Primers | | | | CYP6P3 Primers | | |  |  | Control |
| Replicate | Tech 1 | Tech 2 | Tech 3 | mean |  | Tech 1 | Tech 2 | | Tech 3 | mean | ΔCT |
| 1 | 24.05 | 24.79 | 24.37 | 24.40 |  | 26.67 | 26.66 | | 26.96 | 26.76 | -2.36 |
| 2 | 18.97 | 19.41 | 19.32 | 19.23 |  | 25.68 | 25.70 | | 25.67 | 25.68 | -6.45 |
| 3 | 21.10 | 21.49 | 21.66 | 21.42 |  | 24.80 | 25.63 | | 25.77 | 25.40 | -3.98 |
|  |  |  |  |  |  |  |  | |  | mean | -4.26 |
|  |  |  |  |  |  |  |  | |  | **ΔΔCT** | **4.34** |
