# Supplementary material for: CYP6 P450 Enzymes and ACE-1 Duplication Produce Extreme and Multiple Insecticide Resistance in the Malaria Mosquito Anopheles gambiae
Source: PLoS Genet. 2014 Mar 20;10(3):e1004236. doi: 10.1371/journal.pgen.1004236 (PMC3961184; doi:10.1371/journal.pgen.1004236)
Supplement: Table S4 — GLM testing factors effecting bioassay mortality. (DOCX) [file pgen.1004236.s010.docx]

**Table S4.** Generalised linear model testing the effects of insecticide type, season and PBO on bioassay mortality

| Model terms included | χ^2^ | d.f. | | | P-value | |
| --- | --- | --- | --- | --- | --- | --- |
| insecticide | 5353.2 | 4 | | | ≈ 0 | |
| PBO | 2101.2 | 1 | | | ≈ 0 | |
| season | 0.6 |  | | | 0.42 | |
| insecticide x PBO | 902.7 | 4 | | | ≈ 0 | |
| season x PBO | 12.7 | 1 | | | 0.0004 | |
| insecticide x season | 5.0 | 4 | | | 0.29 | |
| insecticide x season x PBO | 65.1 | 4 | | | 2 x 10^-13^ | |
|  |  | |  |  | |  |

Intercept included in model but not shown (P≈0). Full model shown: removal of the non-significant insecticide x season interaction term had negligible impact on the results.
