# Supplementary material for: CYP6 P450 Enzymes and ACE-1 Duplication Produce Extreme and Multiple Insecticide Resistance in the Malaria Mosquito Anopheles gambiae
Source: PLoS Genet. 2014 Mar 20;10(3):e1004236. doi: 10.1371/journal.pgen.1004236 (PMC3961184; doi:10.1371/journal.pgen.1004236)
Supplement: Table S5 — Resistance association of the G119S target site mutation. (DOCX) [file pgen.1004236.s011.docx]

**Table S5**. Resistance association of the G119S target site mutation, in the presence and absence of PBO following 60 min bendiocarb exposure.

|  |  | *ACE-1* G119S genotype | | | χ^2^ | P |
| --- | --- | --- | --- | --- | --- | --- |
|  |  | S/S | G/S | G/G |  |  |
| bendiocarb | alive | 0 | 49 | 0 | 43.46 | 10^-12^ |
|  | dead | 0 | 12 | 25 |  |  |
|  |  |  |  |  |  |  |
| bendiocarb + PBO | alive | 0 | 38 | 1 | 3.07 | 0.08 |
|  | dead | 0 | 35 | 7 |  |  |
|  |  |  |  |  |  |  |
|  |  |  | Homogeneity test | | 8.28 | 0.004 |
